# Betulinaldehyde exhibits effective anti-tumor effects in A549 cells by regulating intracellular autophagy

**DOI:** 10.1038/s41598-023-27580-w

**Published:** 2023-01-13

**Authors:** Pan-hao Huang, Xiang-bing Duan, Zi-zhao Tang, Zhen-xing Zou, Wen-min Song, Ge Gao, Dai Li, Fang-qin Nie, Xin Yan, Yang-xia Fu, Ren Guo, Yan-ying Xu

**Affiliations:** 1 Department of Pharmacy, The Third Xiangya Hospital, Central South University, Changsha, 410013 Hunan China; 2 Center of Clinical Pharmacology, The Third Xiangya Hospital, Central South University, Changsha, 410013 Hunan China; 3 Department of Laboratory Medicine, The Third Xiangya Hospital, Central South University, Changsha, 410013 Hunan China; 4 Department of Medical Laboratory Science, Xiangya Medical School, Central South University, Changsha, 410013 Hunan China; 5 Phase I Clinical Research Center, Xiangya Hospital, Central South University, Changsha, 410005 Hunan China; 6 Xiangya School of Pharmaceutical Sciences, Central South University, Changsha, 410078 Hunan China; 7 Department of Cardiovascular Medicine, Xiangya Hospital, Central South University, Changsha, 410008 Hunan China

**Keywords:** Cancer, Cell biology

## Abstract

It is of great significance to find new effective drugs for an adjuvant therapy targeting lung cancer to improve the survival rate and prognosis of patients with the disease. Previous studies have confirmed that certain Chinese herbal extracts have clear anti-tumor effects, and in our preliminary study, betulinaldehyde was screened for its potential anti-tumor effects. The current study thus aimed to confirm the anti-tumor effect of betulinaldehyde, using in vitro experiments to explore its underlying molecular mechanism. It was found that betulinaldehyde treatment significantly inhibited the viability, proliferation, and migration of A549 cells in a dose-dependent manner. In addition, betulinaldehyde inhibited the activation of Akt, MAPK, and STAT3 signaling pathways in A549 cells in a time-dependent manner. More importantly, betulinaldehyde also decreased the expression level of SQSTM1 protein, increased the expression level of LC3 II, and increased the autophagy flux in A549 cells. The pretreatment of A549 cells with the autophagy inhibitor, 3-methyladenine, could partially negate the anti-tumor effects of betulinaldehyde. These findings suggest that betulinaldehyde could significantly inhibit the oncological activity of A549 cells by regulating the intracellular autophagy level, making it a potentially effective option for the adjuvant therapy used to treat lung cancer in the future.

## Introduction

Lung cancer is a worldwide malignant disease that causes serious damage to the human body and is the leading cause of death among all forms of cancer^1^. Lung adenocarcinoma, a type of non-small cell lung cancer, is one of the most common types of lung cancer, accounting for about 40% of the diagnosed cases. Compared with small cell lung cancer, it has unique tumor biological characteristics and different prognoses^2^. The pathogenesis of lung adenocarcinoma is complex, involving multiple mutations of proto-oncogenes and the activation of various intracellular molecular signaling pathways^3^. Therefore, an in-depth study of the pathogenesis of lung adenocarcinoma and the search for new therapeutic targets for lung adenocarcinoma to improve the attendant chemotherapy sensitivity will greatly improve the prognosis of patients with the disease and reduce the economic burden on both society and the patients.

The application of traditional Chinese medicine in clinical diseases has made a great contribution to improving the health of people all over the world, with the use of artemisinin in the treatment of malaria and arsenic trioxide in the treatment of leukemia, as well as other major achievements in recent years, confirming the potential efficacy of this type of treatment^4–7^.

In a previous study, our research group extracted a large number of effective components of traditional Chinese medicine monomers that have been proven to be effective in clinical applications. A number of extracts of traditional Chinese medicine with clear anti-cancer activity were then screened via high-throughput screening in the early stage of our research.

Among the screened extracts, betulinaldehyde particularly attracted our attention due to its strong inhibitory effect on A549 cells.

There exist few studies on the role of betulinaldehyde. Pooi Yin Chung et al. demonstrated that, as an FtsZ protein inhibitor, betulinaldehyde can rapidly inhibit the growth of *Staphylococcus aureus* and can play a bactericidal role, while it has synergistic effects with other antibacterial drugs^8^. In addition, studies have also confirmed that betulinaldehyde is a better inhibitor of alpha (α)-glucosidase^9^. Nevertheless, substantial experimental data on the anti-tumor effect of this natural product are still lacking.

Therefore, in this study, the tumor suppressive effect of betulinaldehyde is verified through a variety of molecular biological experiments, with the main molecular signaling pathways involved also determined. Most notably, this study confirms that the tumor-suppressive effect of betulinaldehyde was partly dependent on the regulation of autophagy in A549 cells.

Compared with normal cells, tumor cells have significantly different levels of nutrients and energy metabolisms, and the autophagy, as an important regulatory point for regulating the nutrition and energy supply in cells, is often in an abnormal state in tumor cells^10^. Overall, this study demonstrates that betulinaldehyde could significantly inhibit the oncological activity of A549 cells by regulating the level of intracellular autophagy, thus potentially presenting an effective adjuvant therapy for lung cancer in the future.

## Materials and methods

### Materials

3-Methyladenine (Cat No: HY-19312) and Betulinaldehyde (Cat No: HY-N0084) were purchased from MCE (Shanghai, China). The rAd-mCherry-GFP-LC3B was obtained from Servicebio (Wuhan, China). The anti-AKT (Cat No: 10176-2-AP, 1:1000), anti-AKT-phosphor-Ser473 (Cat No: 66444-1-Ig, 1:3000), anti-SQSTM1 (Cat No: 18420-1-AP, 1:2000), anti-actin (Cat No: 66009-1-Ig, 1:5000), HRP goat anti-Mouse IgG (Cat No: SA00001-1, 1:5000), HRP goat anti-rabbit IgG (Cat No: SA00001-1, 1:6000) were purchased from Proteintech (Hubei, Wuhan, China). The anti-ERK (Cat No: ab184699, 1:10000) was purchased from abcam (Cambridge, UK). Anti-phosphor-ERK (Cat No: AF1015, 1:1000) was purchased from affbiotech (Cincinnati, OH, USA). The anti-LC3 (Cat No: 12741S, 1:1000) was purchased from Cell Signaling Technology (Massachusetts, USA). Anti-phosphor-STAT3-Tyr705 (Cat No: abs130918, 1:1000) was bought from absin (China). And anti-STAT3 (Cat No: GB11176, 1:1000) was obtained from Servicebio (Wuhan, China).

### Animals

Nude mice were obtained from Hunan SJA Laboratory Animal Co., Ltd. All animal (female, n = 28, age 4–6 weeks) were acclimatized one week prior to the start of the study and housed in a standard facility with 12 h light–dark cycles and controlled temperature (21–22 °C). The mice were fed a standard pellet chow and water. All animal studies were conducted according to the National Institutes of Health Guide for the Care and Use of Laboratory Animals, and followed the recommendations in the ARRIVE guidelines. The study was approved from ethics committee of Department of Laboratory animal of Central South University.

### Cell culture

Human A549 (Cat No: ZQ0003) were abtained from Zhongqiaoxinzhou Biotech (Shanghai, China). A549 cells were cultured A549 in DMEM (4500 mg/L D-Glucose, Sigma, Cat No: D5796) supplemented with 10% FBS. Cells were incubated at 37 °C with CO_2_ set to 5%.

### In vivo xenograft model

Betulinaldehyde was dissolved in DMSO to form a 20 mg/mL reserve solution. After a week of adaptive feeding, 2 × 10^6^/100 μL non-small-cell Lung Carcinoma Cells A549 were injected subcutaneously into the armpit of nude mice. When the tumor was injected 14 days later, the mice were randomly divided into 4 groups, 7 nude mice in each group, respectively as control group (DMSO), Betulinaldehyde-L (50 mg/kg), Betulinaldehyde-M (100 mg/kg), and Betulinaldehyde-H (200 mg/kg) were intraperitoneally injected in nude mice, the drug was administered once a day for a week.

### Determination of tumor growth curve and tumor inhibition rate

Tumor size was measured on the long axis (A) and short axis (B) with vernier caliper, and the calculation formula was V(CM3) = A × B2/2. After 21 days of tumor implantation, the nude mice were sacrificed by cervical dislocation.

### Immunohistochemistry

Transplanted A549 tumor tissue was washed with normal saline, then soaked with 4% paraformaldehyde solution, fixed for 24 h, then routinely dehydrated, transfused and embedded in paraffin after fishing slice, 60 °C baking slice for 12 h, put into the slice box and put in 4 °C refrigerator to keep for reserve. After the wax removal was completed, the sectioned slices were incubated with endogenous peroxidase blocker for 10 min and goat serum for 1 h. Then the slices were incubated with anti-Ki67 and anti-VEGF antibodies overnight at 4 °C. Subsequently, the sections were washed three times, and further incubated with the goat anti-rabbit secondary antibody. Ultimately, the sections were incubated with DAB, and the cells were observed and photographed by light microscope.

### TUNEL staining

TUNEL assay method was used to detect apoptotic cells in tumor tissues. Tissue sections were prepared as described in immunohistochemistry, the deparaffinized and rehydrated tumor sections were permeabilized with proteinase K for 30 min, washed with PBS, and then treated with TUNEL assay kit (NanJing KeyGen Biotech, China) according to the manufacturer's instructions. After the sections were developed by DAB chromogenic solution and counterstained with hematoxylin, the excess liquid was washed with ddH_2_O. At last, the TUNEL positive cells were observed and photographed with light microscope after adding neutral gum and cover glass slide.

### Western blotting

Total proteins of A549 were extracted and cultured cells were lysed in RIPA buffer. Proteins were separated by 10% SDS-PAGE (Beyotime Biotech, China) and then transferred onto a nitrocellulose filter (NC) (Pall, USA) membrane after electrophoresis. After 1 h blocking in 5% skim milk, the membranes were immunoblotted with primary antibodies overnight at 4 °C, followed by secondary antibodies for 1 h at room temperature. The immunoblots were detected using a Bio-Rad ChemiDoc system (Hercules, CA). The blots cut prior to trarsmembran according to the different molecular weight in order to save antibodies, and then hybridisation with antibodies, so we unable to provide images showing full length membranes. Images of all blots and all replicates (repeat three times) showed in the Supplementary material. The images were collected from different parts of the same gel with same loading amounts.

### CCK-8 assays for cell viability

A549 cells were seeded to 96-well plate and incubated for 24 h at 37 °C with CO_2_ set to 5%. After different means of treatment, CCK-8 solutions were prepared and added to each well, and then incubated at 37 °C for 4 h with 5% CO_2_. The absorbance (OD) at 450 nm was analyzed by Heales MB-530 microplate analyzer (Shenzhen, China), repeat the analysis three times and take the mean value as the histogram.

### Colony formation assay

A549 cells (200/well) were seeded to 6-well cell culture plates. After 2–3 weeks treatment, the medium was aspirated and the cells were rinsed with PBS for 2 times, and then 1 mL 4% paraformaldehyde was added to each well to fix the cells. After fixation for 15 min, the cells were washed with PBS twice. Then cells were treated with crystal violet staining assay for 30 min to make them visible. The wells were rinsed carefully with clean water and then air-dried at room temperature. The whole image of the plate was obtained by a camera. The OD value of wells was detected for 3 times using the Heales MB-530 microplate analyzer (Shenzhen, China) at the wavelength of 550 nm.

### Measurement of the migration of A549

A549 were seeded to the 6-well cell culture plate. After the bottom of the culture plates were covered with monolayer cells, make scratches in each well by using the sterile tip, and then washed with PBS three times. Measurement of the migration after wounding of A549 was detected by using Operetta High Content Cytometers (Perkin Elmer, America) analysis.

### EdU assay

A549 cells were seeded to 6-well plate. After treatment with corresponding methods, added prepared 50 μmol/L EdU work solution to each well. Fix cells for 30 min with 4% Polyformaldehyde, and then added PBS solution containing 0.5% Tritonx-100 to the cell culture plate, incubated cells on a shaker for 10 min, and washed the plate with PBS for 5 min. Then the cells were incubated with Apollo® staining solution and Hoechst 33342 solution for 30 min respectively on a shaker without light. The samples were detected by Operetta High Content Cytometers (Perkin Elmer, America).

### rAd-mCherry-GFP-LC3B transfection assay

A549 cells were seeded onto an 12-well cell culture plates at 5 × 10^5^ cells/well and incubated at 37 °C with CO_2_ set to 5% for 24 h. After the cells adhered to the wall, the cells were transfected with rAd-mCherry-GFP-LC3B adenovirus (HonorGene, China) at multiplicity of infection (MOI) of 100, and then incubated at 37 °C in a 5% CO_2_ environment for 48 h. The autophagy puncta was identified by using Zeiss LSM 880 confocal laser scanning microscopes (Zeiss, Germany). In the green and red merged images, the yellow and red puncta indicated autophagosomes and autophagolysosomes, respectively.

### Statistical analysis

Spss24.0 software (IBM, USA) was used for statistical analysis. All data were expressed as the mean ± SD. Each experiment was repeated at least 3 times. Statistical significance was evaluated by one-way analysis of variance followed by LSD for post hoc test. *P* < 0.05 was considered to be statistically significant.

## Results

### Inhibition of the viability and colony formation of A549 cells by betulinaldehyde

**Figure 1:**
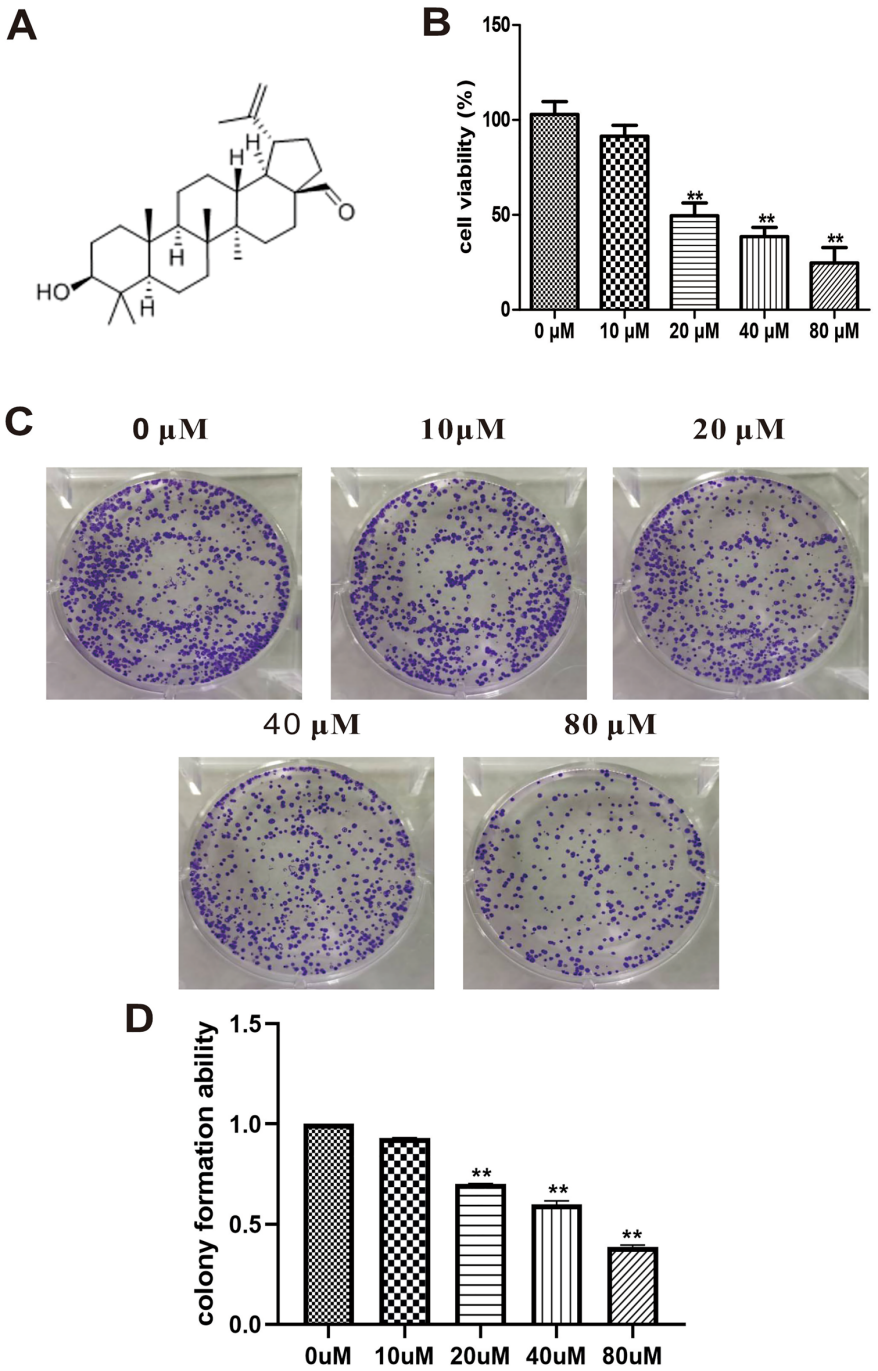
Betulinaldehyde inhibited the viability and colony formation of A549 cells. (**A**) The chemical structure of betulinaldehyde. (**B**) A549 cells were treated with different concentrations of betulinaldehyde for 24 h and the cell viability was detected via CCK8. (**C**,**D**) The colony formation ability was detected via crystal violet staining. All values are expressed as the mean ± SD (*n* = 3). Statistical significance was evaluated by one-way analysis of variance followed by LSD for post hoc test. **P < 0.01 versus 0 μM group.

After the potential tumor-inhibition effect of betulinaldehyde was identified in the previous screening test (Fig. 1A), the A549 cells were further treated with different concentrations of the compound to determine its tumor inhibition effect in vitro. As shown in Fig. 1B, the analysis of the cell counting Kit-8 (CCK8) data revealed that 10 μM of betulinaldehyde had no significant effect on cell viability; however, when the concentration exceeded 20 μM, the A549 cells activity was significantly inhibited compared with the control group. Furthermore, when the concentration reached 80 μM, the activity of A549 cells was completely inhibited. Similar to the CCK8 experiment, the results of colony formation experiment also suggested that betulinaldehyde could inhibit the colony formation ability of A549 cells in a concentration-dependent manner. As shown in Fig. 1C and D, the positive colony number of A549 cells was reduced by betulinaldehyde concentrations of > 20 μM, while the colony formation ability of the A549 cells was almost completely inhibited when the concentration reached 80 μM.

### The effect of betulinaldehyde on the intracellular signaling pathways of A549 cells

**Figure 2:**
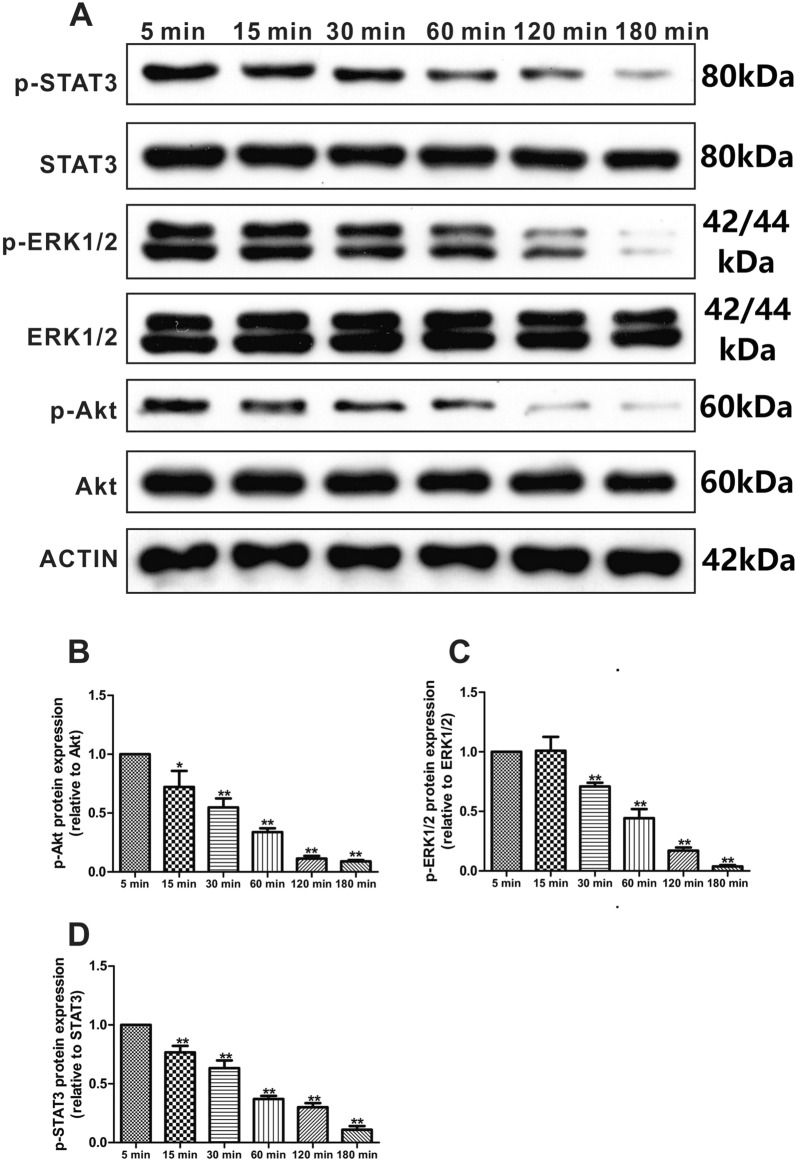
The effect of betulinaldehyde on the intracellular signaling pathways of A549 cells. (**A**) A549 cells were treated with 20-μM betulinaldehyde at different time points before the cell extracts were analyzed using the western blotting technique. The statistical analyses of (**B**) p-Akt, (**C**) p-ERK1/2, and (**D**) p-STAT3 are included. Cropped images are displayed, uncropped blots are displayed in Supplementary Fig. 1. The images were collected from different parts of the same gel with same loading amounts. The blot gels shown are representatives of three different experiments (n = 3). All values are expressed as the mean ± SD (*n* = 3). Statistical significance was evaluated by one-way analysis of variance followed by LSD for post hoc test. *P < 0.05, **P < 0.01 versus 5 min group.

After determining the anti-tumor effect of betulinaldehyde on A549 cells, we further explored the effect of betulinaldehyde on the intracellular signaling pathways of A549 cells by means of molecular biological experiments. Here, the main focus was on the effect of betulinaldehyde on protein kinase B (Akt), mitogen‑activated protein kinase (MAPK), and signal transducer and activator of transcription 3 (STAT3), three signaling pathways known to play an important role in tumorigenesis and development. Since betulinaldehyde has a significant anti-tumor effect at 20 μm, at this concentration, we analyzed changes in the levels of related proteins in the intracellular signaling pathways at different time points to test whether it has time-dependent pharmacological effects. Following the treatment of the A549 cells with 20-μM betulinaldehyde for 60 min, the phosphorylated Akt level was significantly inhibited, while the activity of the Akt signaling pathway was almost completely inhibited after 180 min (Fig. 2A,B). As shown in Fig. 2C and D, 20-μM betulinaldehyde also demonstrated a clear inhibitory effect on the phosphorylation level of the extracellular signal-regulated kinase (ERK1/2) and STAT3 pathways at 120 and 60 min, respectively, reaching the peak effect at 180 min. These results confirm that betulinaldehyde may play an anti-tumor role in vitro through extensive inhibition of multiple intracellular signaling pathways.

### The effect of betulinaldehyde on the intracellular autophagy level of A549 cells

**Figure 3:**
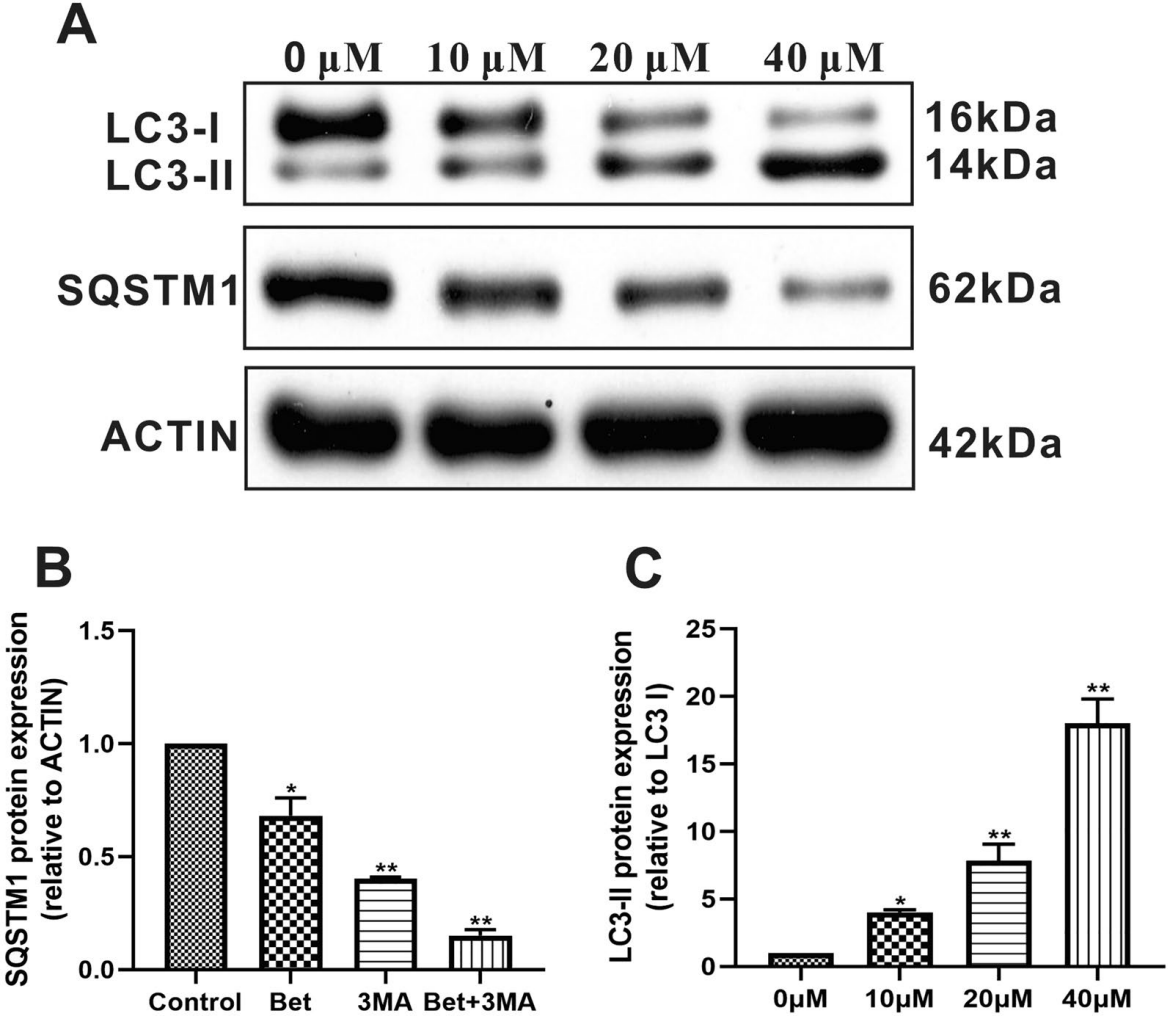
The effect of betulinaldehyde on the SQSTM1 and LC3-II levels of A549 cells. (**A**) A549 cells were treated with different concentrations of betulinaldehyde for 24 h before the cell extracts were analyzed using the western blotting technique. The statistical analyses of the (**B**) SQSTM1 levels and (**C**) LC3-II levels are included. Cropped images are displayed, uncropped blots are displayed in Supplementary Fig. 2. The images were collected from different parts of the same gel with same loading amounts. The blot gels shown are representatives of three different experiments (n = 3). All values are expressed as the mean ± SD (*n* = 3). Statistical significance was evaluated by one-way analysis of variance followed by LSD for post hoc test. *P < 0.05, **P < 0.01 versus 0 μM group.

The intracellular autophagy level often changes according to the variation in the cell’s external environment and nutrition supply. Therefore, the level of autophagy in tumor cells is often comparatively different from that in normal cells. The Akt signaling pathway is recognized as a molecular signaling pathway that can significantly influence the level of autophagy. The suppression of the Akt signaling pathway by betulinaldehyde led us to further explore the latter’s possible regulation of the intracellular autophagy process. As shown in Fig. 3A–C, compared with the control group, the A549 cells treated with different concentrations of betulinaldehyde for 24 h significantly regulated the marker proteins of autophagy, as demonstrated by the dramatic down-regulation of the SQSTM1 protein level and the significant increase in the LC3-II protein level.

### The effect of betulinaldehyde on the autophagy flux of A549 cells

**Figure 4:**
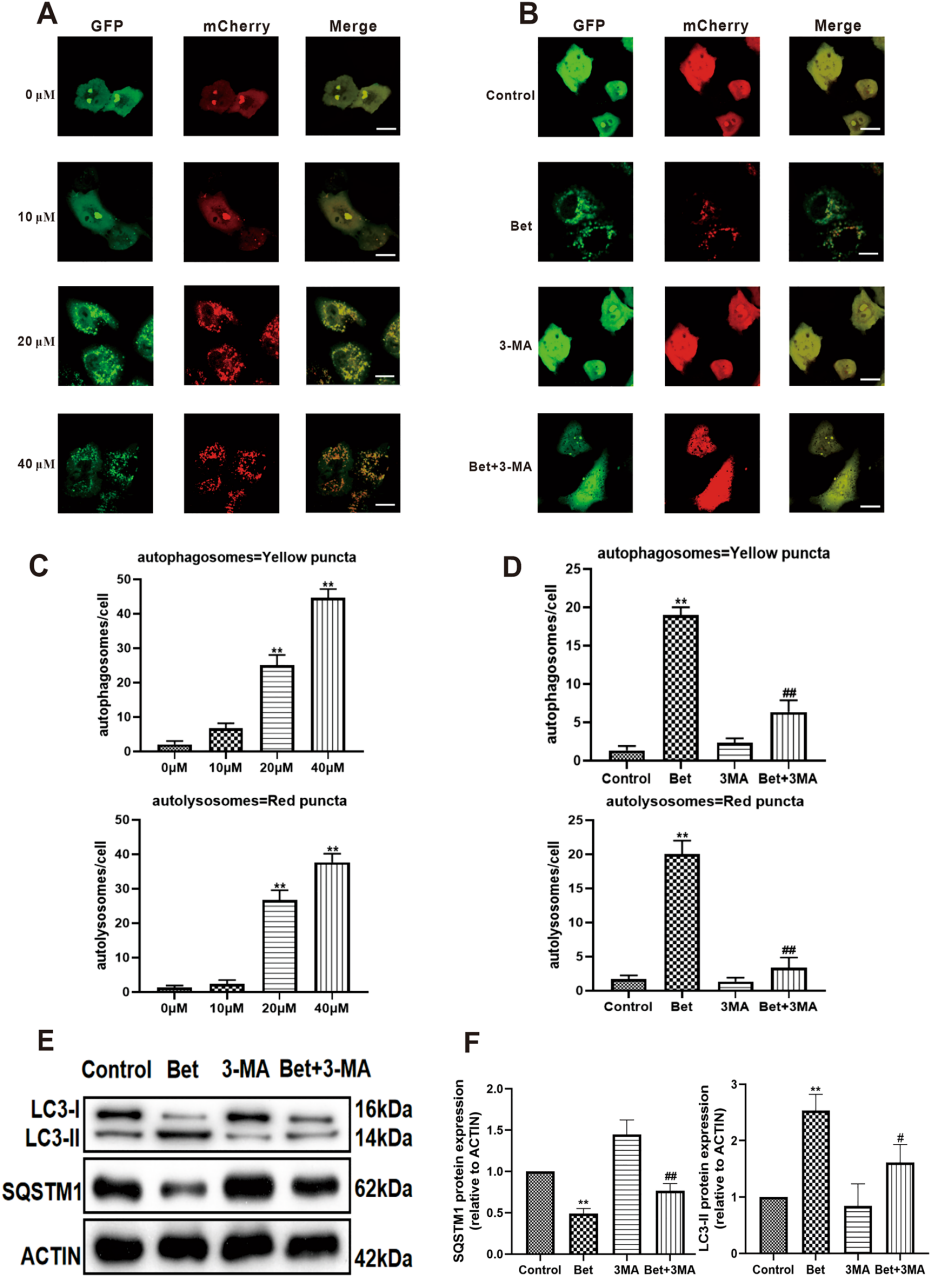
Betulinaldehyde promoted the autophagy flux in A549 cells. (**A**) A549 cells were treated with different concentrations of betulinaldehyde for 24 h before the autophagy flux was analyzed via a confocal laser scanning microscope using mCherry-GFP-LC3B. (**B**) 3-MA was also used to determine the effects of 20-μM betulinaldehyde on the autophagy flux in A549 cells. (**C**,**D**) Quantification of LC3B positive autolysomomes or autophagosomes in A549/mCherry-GFP-LC3B cells. (E) Expressions of SQSTM1 and LC3-II were tested at the protein level by western blotting. The statistical analyses of the (F) SQSTM1 levels and LC3-II levels are included. Cropped images are displayed, uncropped blots are displayed in Supplementary Fig. 3. The images were collected from different parts of the same gel with same loading amounts. The blot gels shown are representatives of three different experiments (n = 3). All values are expressed as the mean ± SD (*n* = 3). Comparisons among multiple groups were performed using one-way analysis of variance. The differences between control and other groups were analyzed using LSD multiple comparison test. *P < 0.05, **P < 0.01 versus control group, ^#^*P* < 0.05, ^##^*P* < 0.01 versus Bet group (Bet = betulinaldehyde, 3-MA = 3-methyladenine). Scale bar, 20 μm.

With the in-depth study of the autophagy, it was found that the simple detection of LC3-II and SQSTM1 levels could only reflect the induction of the autophagy level and the autophagosome clearance. In fact, a comprehensive evaluation of the autophagy should not only include the detection of the autophagosome level, but should also include determining whether the whole autophagy flux process is smooth. Therefore, in this study, the effect of betulinaldehyde on the autophagy flux in A549 cells was examined using an Ad-mCherry-GFP-LC3B experiment. As expected, with the increase in betulinaldehyde concentration, the number of autophagosomes in A549 cells increased continuously, indicating an increase in intracellular autophagy flux (Fig. 4A). In addition, following treatment with 20-μM betulinaldehyde for 24 h, the autophagy flux in the A549 cells was further increased, while the SQSTM1 protein level was significantly down-regulated and the LC3-II protein level was significantly increased. Meanwhile, following pretreatment with 5 mM autophagy inhibitor 3-methyladenine (3-MA) for 30 min, then cotreated with 20-μM betulinaldehyde for 24 h, the autophagic flux induced by the betulinaldehyde was disrupted, which suggested that the SQSTM1 protein level was up-regulated and the LC3-II protein level was reduced (Fig. 4B–F). Together, these results indicate that betulinaldehyde promotes the autophagy flux in A549 cells.

### Inhibition of the viability and proliferation of A549 cells by betulinaldehyde via regulation of the autophagy level

**Figure 5:**
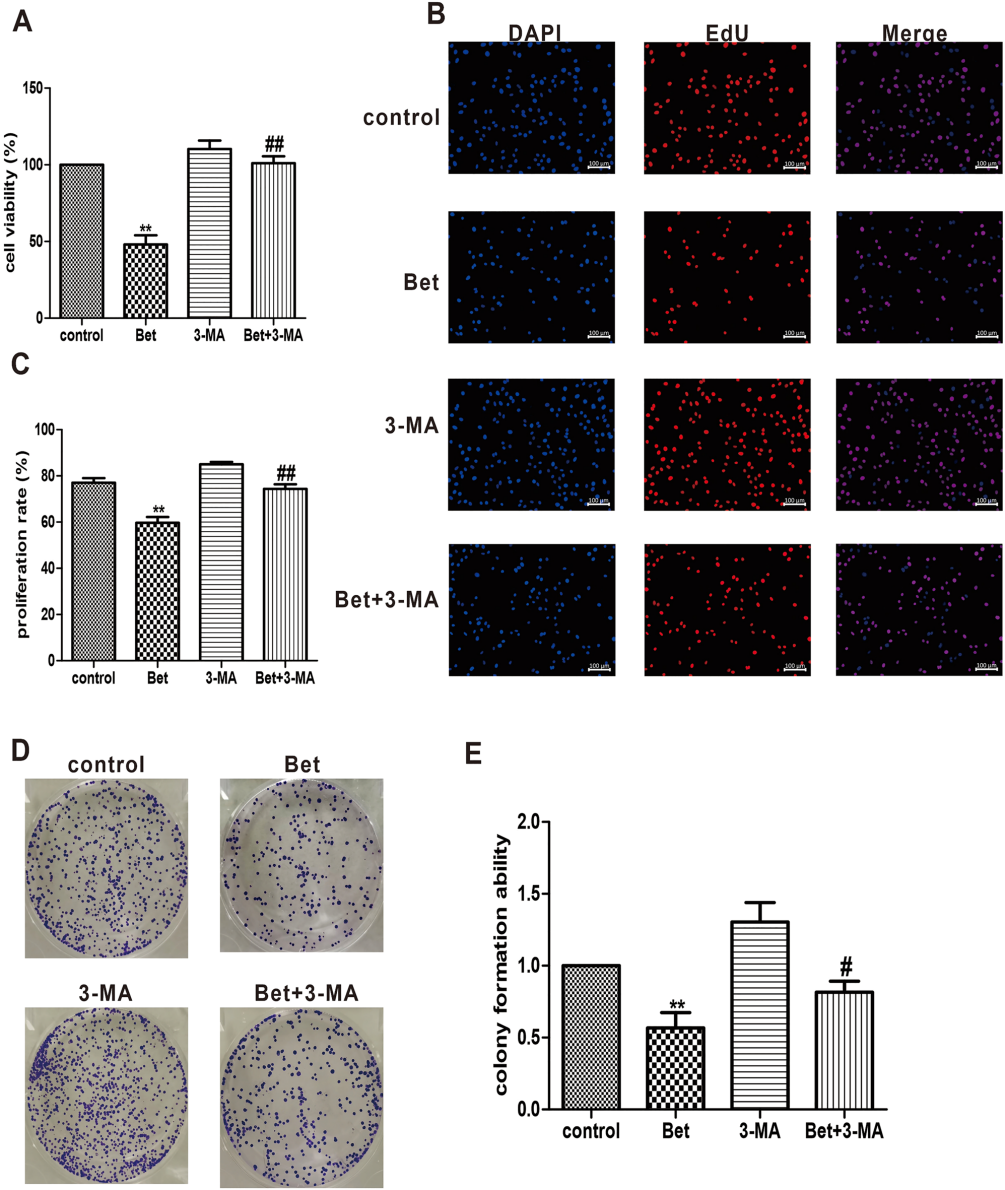
Autophagy mediated the effect of betulinaldehyde on viability and proliferation of A549 cells. (**A**) A549 cells were treated with 20-μM betulinaldehyde for 24 h with or without 3-MA before the cell viability was determined via CCK8. (**B**) The cell proliferation was measured via EdU staining. (**C**) The statistical analyses of the proliferation ability. (**D**,**E**) Colony formation of the A549 cells analyzed via crystal violet staining. All values are expressed as the mean ± SD (*n* = 3). Comparisons among multiple groups were performed using one-way analysis of variance. The differences between control and other groups were analyzed using LSD multiple comparison test. **P < 0.01 versus control group, #*P* < 0.05, ##*P* < 0.01 versus Bet group (Bet = betulinaldehyde, 3-MA = 3-methyladenine). Scale bar, 100 μm.

To further analyze whether the anti-tumor effect of betulinaldehyde is related to its autophagy regulation, 20-μM betulinaldehyde was used to treat the A549 cells for 24 h with or without 3-MA before determining the cell viability, proliferation, and colony formation. As shown in Fig. 5A–E, the viability of A549 cells was significantly decreased following 20-μM betulinaldehyde treatment for 24 h, while the number of ethynyldeoxyuridine (EdU) positive cells and the colony formation ability were also significantly decreased when compared to the control group. However, these effects of betulinaldehyde could be partially negated by the pretreatment of A549 cells with 3-MA.

### Inhibition of the migration of A549 cells by betulinaldehyde via regulation of the autophagy level

**Figure 6:**
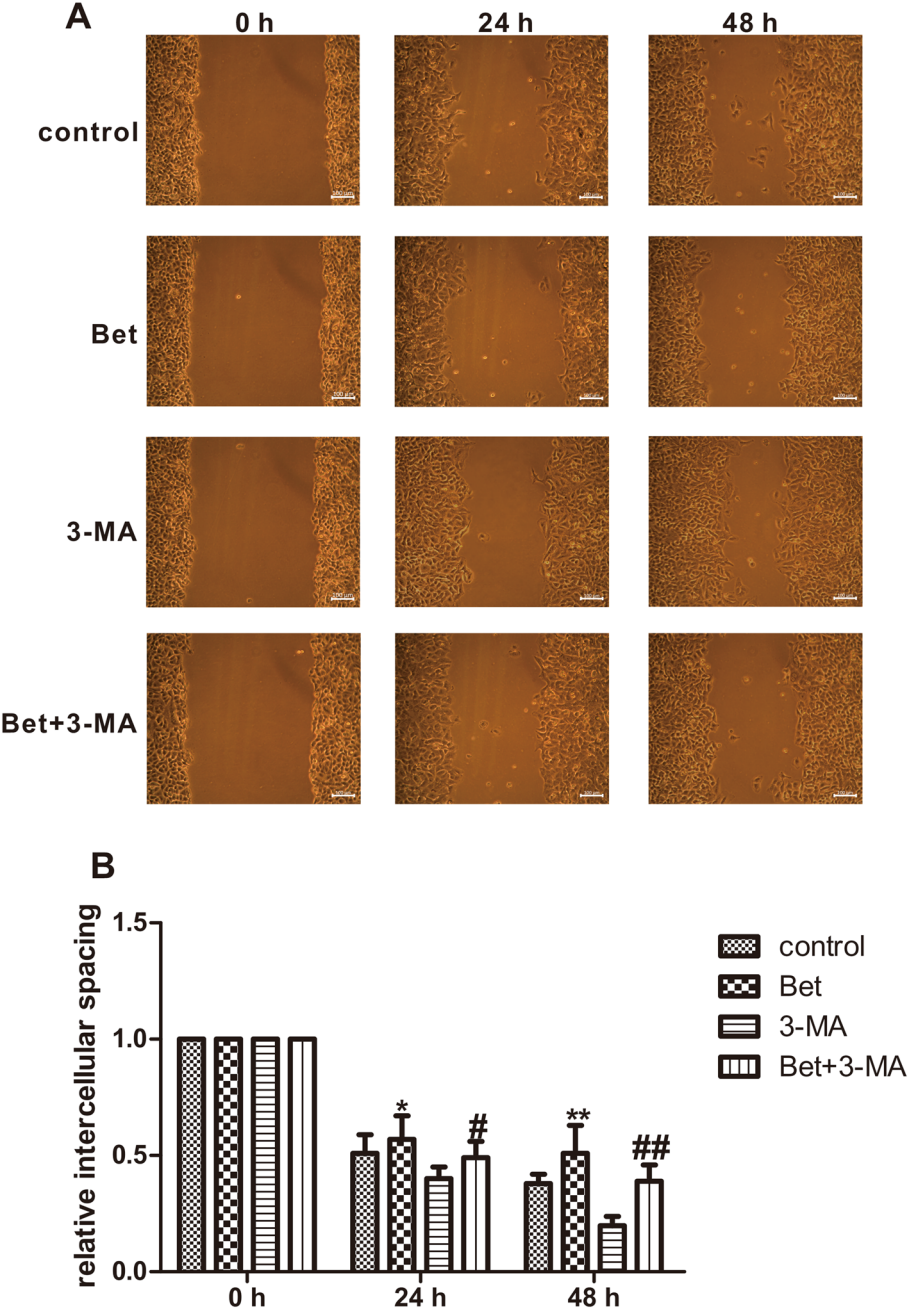
Autophagy mediated the effect of betulinaldehyde on migration of A549 cells. (**A**) A549 cells were treated with betulinaldehyde (20 μM) for 24 h, with or without 3-MA, and the intercellular spacing was observed at different time points. (**B**) The statistical analyses of the migration ability. All values are expressed as the mean ± SD (*n* = 3). Statistical significance was evaluated by one-way analysis of variance followed by LSD for post hoc test. *P < 0.05, **P < 0.01 versus control group, ^#^*P* < 0.05, ^##^*P* < 0.01 versus Bet group (Bet = betulinaldehyde, 3-MA = 3-methyladenine). Scale bar, 100 μm.

The possible effect of autophagy on the betulinaldehyde-inhibition of A549 cells migration was also explored. As shown in Fig. 6A and B, compared with the control group, 20-μM betulinaldehyde significantly inhibited the migration of A549 cells, exhibiting a larger intercell spacing between separated cells. However, if A549 cells were treated with 3-MA in advance, the inhibition of A549 cells migration could be partially eliminated.

### The inhibitory effect of betulinaldehyde on in vivo carcinogenesis of lung cancer

**Figure 7:**
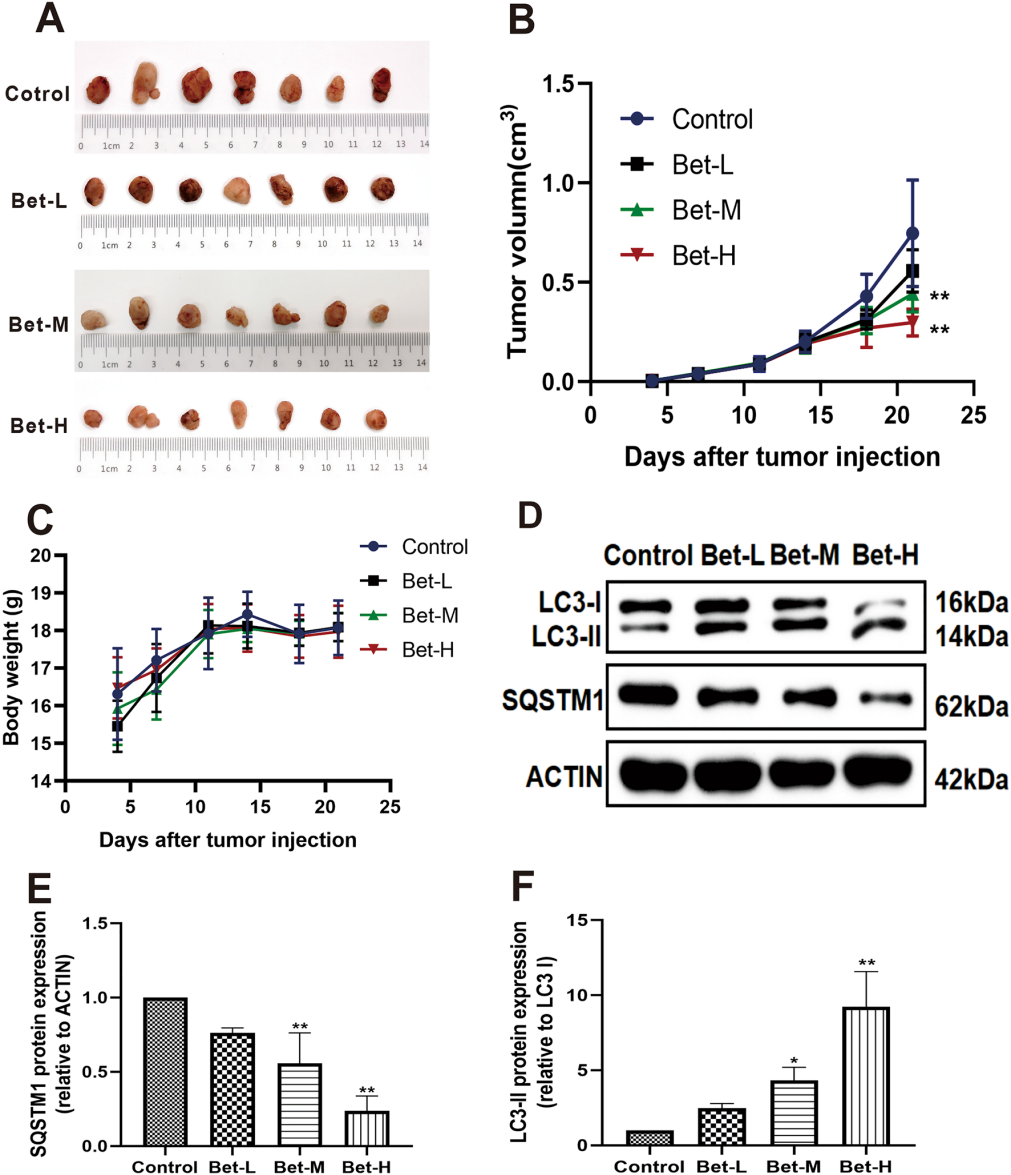
Betulinaldehyde inhibited the growth of A549 tumor xenografts in nude mice in vivo. After adaptive feeding for one week, A549 cells were implanted into nude mice via subcutaneous injection under the axilla, and the mice were treated with different concentrations of betulinaldehyde when the tumor grew to a suitable size. (**A**) The xenograft tumors were separated when the animals were killed. (**B**) The tumor growth was measured every 4 days. Results were presented as mean ± SD (n = 7). (**C**) The total body weight was monitored for 21 days. (**D**–**F**) The expressions of SQSTM1 and LC3-II were tested at the protein level and the data were quantitated to be represented as mean ± SD (n = 3). Cropped images are displayed, uncropped blots are displayed in Supplementary Fig. 4. The images were collected from different parts of the same gel with same loading amounts. The blot gels shown are representatives of three different experiments (n = 3). Comparisons among multiple groups were performed using one-way analysis of variance. The differences between control and treatment groups were analyzed using LSD multiple comparison test. *P < 0.05, **P < 0.01 versus control group, ^#^*P* < 0.05, ^##^*P* < 0.01 versus Bet group (Bet-L = betulinaldehyde [50 mg/kg]; Bet-M = betulinaldehyde [100 mg/kg]; Bet-H = betulinaldehyde [200 mg/kg].

The anti-tumor effect of betulinaldehyde was further investigated in vivo using a subcutaneous tumor transplantation model. As shown in Fig. 7A, different doses of betulinaldehyde significantly reduced the size of the tumor in a dose-dependent manner, with the effect of the high-dose group found to be the best. As shown in Fig. 7B, the growth of the tumor in nude mice, transplanted via subcutaneous tumor implantation, was slowed to a certain extent by different concentrations of betulinaldehyde, with the tumor growth curve exhibiting a flat trajectory. Besides, no significant body weight loss or treatment-related deaths following therapy were observed, which demonstrated that betulinaldehyde suppressing the tumor growth without additional toxicity (Fig. 7C). Similarly, it was also observed that betulinaldehyde significantly up-regulated the autophagy level in tumor tissues, which was evidenced by the down-regulation of SQSTM expression and the up-regulation of LC3-II expression (Fig. 7D–F).

**Figure 8:**
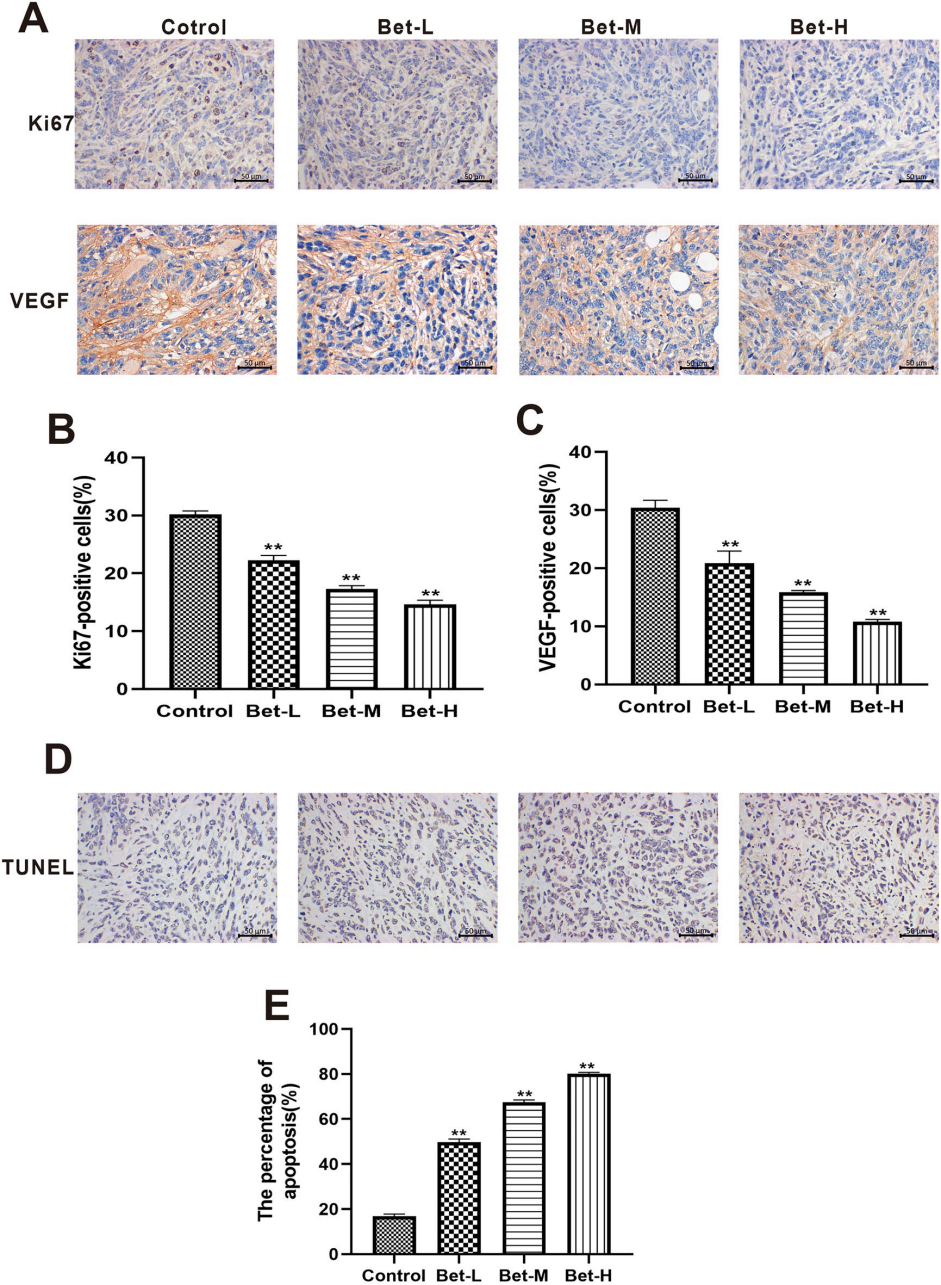
The effect of betulinaldehyde on the expression of proliferation and apoptosis markers in tumor tissues. (**A**) Representative IHC images of VEGF and Ki67 of the tumors. (**B**,**C**) Quantification analysis of immunoreactive cells for VEGF and Ki67. Data are presented as mean ± SD (n = 4). (**D**) TUNEL analysis image at 3 weeks after transplantation. (**E**) Quantified TUNEL positive apoptotic cells count. All values are expressed as the mean ± SD (*n* = 4). Comparisons among multiple groups were performed using one-way analysis of variance. The differences between control and treatment groups were analyzed using LSD multiple comparison test. *P < 0.05, **P < 0.01 versus control group, ^#^*P* < 0.05, ^##^*P* < 0.01 versus Bet group (Bet-L = betulinaldehyde [50 mg/kg]; Bet-M = betulinaldehyde [100 mg/kg]; Bet-H = betulinaldehyde [200 mg/kg]. Scale bar, 50 μm.

The expression of vascular endothelial growth factor (VEGF) is closely related to the generation of blood vessels in tumor tissues and the growth of lung cancer. In short, the increase in VEGF expression can increase the generation of blood vessels in tumor tissues, thereby promoting the growth of lung cancer. Therefore, the VEGF expression was determined using an immunohistochemistry experiment, with the tumorigenic capacities determined based on the proliferation marker (Ki-67) and apoptosis marker (TUNEL). As expected, the betulinaldehyde significantly down-regulated the expression levels of Ki-67 and VEGF in the tumor tissues, inhibited tumor growth, and significantly increased the TUNEL expression level, which promoted the apoptosis of the tumor cells (Fig. 8A–E). These results indicate that betulinaldehyde has an inhibitory effect on in vivo carcinogenesis of lung cancer.

## Discussion

Betulinaldehyde is derived from the dried leaves of *Dillenia turbinata Finet and Gagnep*, a species of tree in the family *Dilleniaceae*. Recent studies have demonstrated that as an α-glucosidase inhibitor, betulinaldehyde has a certain ability to regulate blood glucose and has also demonstrated a certain antibacterial activity in various in vitro studies^11,12^. Feng Yang et al. found that betulinaldehyde is a natural RAR-related orphan receptor gamma (RORγt) agonist, changing the thermal stability of the gene by directly binding to proteins. As the agonists and inhibitors of RORγt are potential drugs for tumor immunotherapy and autoimmune diseases, respectively, as previous studies have indicated, betulinaldehyde may have a certain immunomodulatory effect^13^. Combined with previous high-throughput screening studies, our experimental results confirmed for the first time that betulinaldehyde has a strong inhibitory effect on the tumor activity of A549 cells.

Previous studies lacked an exploration of the anti-cancer effect of betulinaldehyde, meaning the regulation mechanism of betulinaldehyde on the molecular signaling pathway in tumor cells remained unclear. In this study, after determining the anti-tumor effect of betulinaldehyde in a concentration-dependent manner, the influence of betulinaldehyde on several major signaling pathways in A549 cells related to cell proliferation activity was further examined. The intracellular signaling pathways that play a key role in regulating the rapid division and proliferation of tumor cells mainly include PI3K/Akt, MAPK, and JAK/STAT^14,15^. In fact, Akt is a serine/threonine kinase that is recruited by binding to the phosphatidylinositol in the cell membrane when the external environment changes or when intracellular nutrients are sufficient, thus fully activating the pathway. The activated Akt then mediates the downstream reactions by phosphorylating a range of intracellular proteins. For example, activated Akt influences many apoptotic factors through transcriptional regulation or direct phosphorylation. In the nucleus, Akt inhibits transcription factors related to cell death, resulting in decreased expression of cell death genes and enhanced transcription of anti-apoptotic genes^16^. In addition, a number of studies have also demonstrated that the Akt signaling pathway may exert significant regulatory effects on the intracellular autophagy by regulating key factors in autophagy regulation, such as mechanistic target of rapamycin (mTOR) and Forkhead Box O1 genes, and ultimately participates in the occurrence and development of a variety of diseases^17,18^. In line with the observed anti-tumor effect of betulinaldehyde in vitro, the compound could inhibit the phosphorylation level of Akt in A549 cells in a concentration-dependent manner, suggesting its inhibitory effect on the PI3K/Akt pathway.

In addition to the inhibition of the Akt pathway, we also observed that betulinaldehyde had a significant inhibitory effect on the MAPK pathway and the STAT3 pathway, suggesting that it may play an anti-tumor role in terms of regulating multiple signaling pathways in cells. Previous studies have also demonstrated that the activation of the MAPK pathway is related to the remodeling of cytoskeleton protein, which plays an important role in the migration of tumor cells^19^. Therefore, the inhibition of A549 migration by betulinaldehyde may be related to the inhibition of the MAPK signaling pathway.

Tumor cells exhibit stronger cell proliferation ability than normal cells. Thus, in this study, the proliferative cells in A549 cells were labeled using EdU staining to evaluate the effect of betulinaldehyde on the proliferation ability of A549 cells. As expected, the betulinaldehyde significantly inhibited the proliferation of A549 cells. Taken together, these results suggest that betulinaldehyde can not only induce decreased A549 cells activity, but also further plays an anti-tumor role by inhibiting the proliferation ability of A549 cells. The double inhibition of tumor cell activity and proliferation undoubtedly helps betulinaldehyde to play its anti-tumor role more effectively.

Another finding of this study relates to the regulation of the autophagy level in A549 cells by betulinaldehyde, with this regulation partly mediating the tumor-suppressing effect of the compound. The regulation of autophagy by betulinaldehyde may depend on its inhibition of the Akt pathway. Previous studies have proven that an abnormal autophagy level is involved in the occurrence and development of tumor diseases, while there exist numerous conflicting reports on whether autophagy plays an inhibitory or promoting role in such diseases. During the process of tumor development, the PI3K/Akt and Akt/mTOR signaling pathways are activated and p53 protein is accumulated in the cytoplasm, leading to autophagy dysfunction and subsequent instability of the genome, as well as blocked cell differentiation, retarded cell senescence, and disruption of the cell metabolism, ultimately forming a vicious cycle of tumorigenesis promotion^20–22^. However, under certain conditions, autophagy has been proven to promote tumorigenesis. In the late stage of tumor progression, the autophagy level is often active, which enhances the ability of tumor cells to cope with endogenous stress and also increases the resistance of cells to chemotherapy or radiotherapy treatment^23,24^. Overall, the effect of the autophagy level on tumor disease varies according to the disease progression and the changes in the microenvironment of the tumor cells.

In this study, using a loss of function experiment, it was confirmed that part of the anti-tumor effect of betulinaldehyde is mediated through the autophagy pathway, as indicated by the partial negation of the anti-tumor effect of betulinaldehyde by the autophagy inhibitors. Since betulinaldehyde regulates the autophagy level and also has an inhibition effect on α-glucosidase, it may play a certain role in the treatment of certain metabolic diseases, such as obesity, hyperlipidemia, and non-alcoholic fatty liver disease, through metabolism regulation.

In conclusion, our study provides strong experimental evidence that betulinaldehyde, an extract used in traditional Chinese medicine, has a significant anti-tumor effect in vitro and that its anti-tumor mechanism may be related to its inhibition of multiple pro-tumor signaling pathways in tumor cells. In addition, it was demonstrated that the anti-tumor effect of betulinaldehyde was partly dependent on its regulation on the autophagy levels in A549 cells. Based on its inhibition of multiple signaling pathways in tumor cells and its regulation of both autophagy and metabolism, it is expected that betulinaldehyde will be used as an important adjuvant therapy drug in the treatment of tumors.

## Supplementary Information

Supplementary Figure 1.

Supplementary Figure 2.

Supplementary Figure 3.

Supplementary Figure 4.

## Data Availability

The datasets generated during and/or analyzed during the current study are available from the corresponding author on reasonable request.
