# Supplementary figures and images for: Betulinaldehyde exhibits effective anti-tumor effects in A549 cells by regulating intracellular autophagy
Source: Sci Rep. 2023 Jan 13;13:743. doi: 10.1038/s41598-023-27580-w (PMC9839726; doi:10.1038/s41598-023-27580-w)

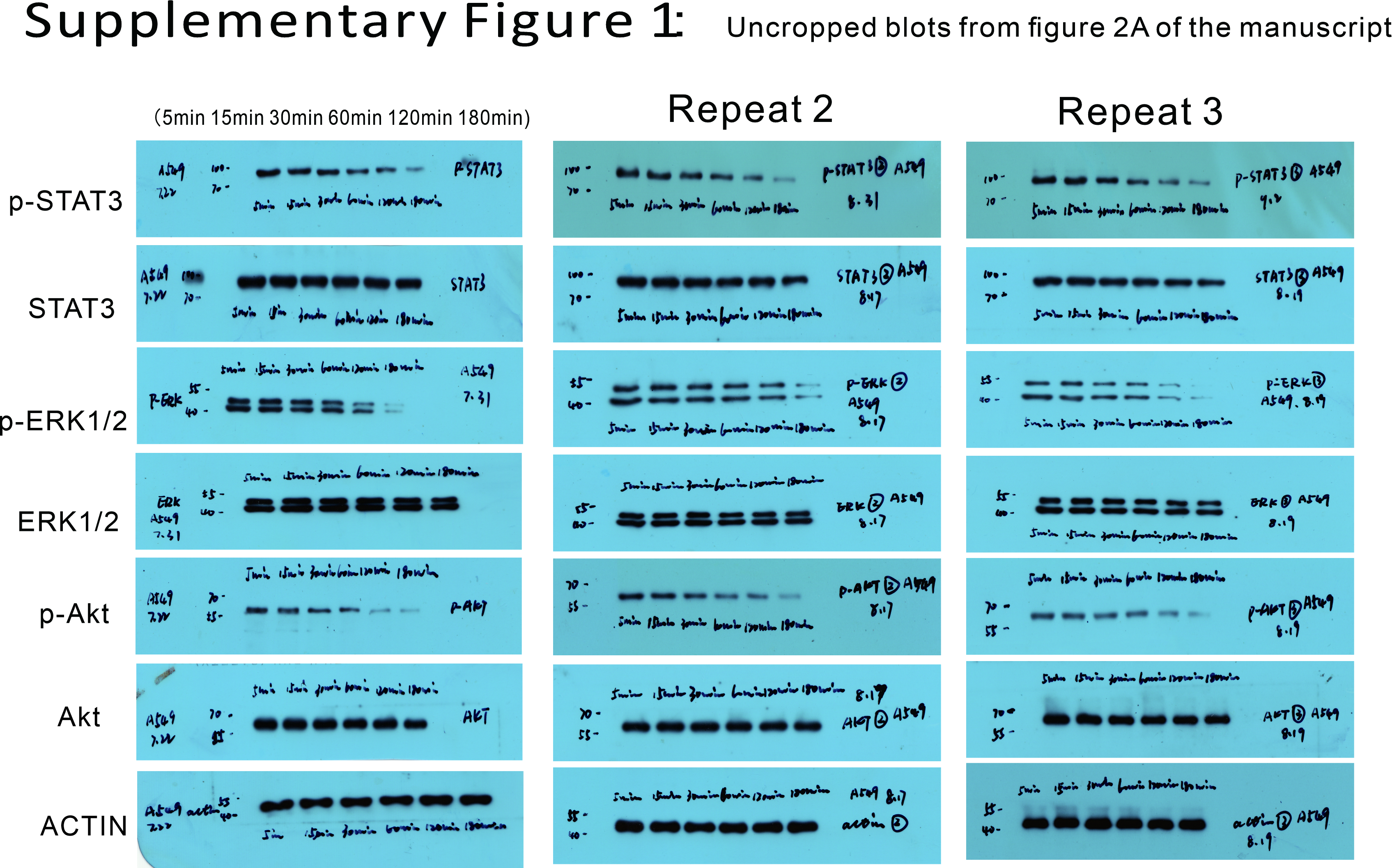

Supplement: Supplementary file 1 — Supplementary Figure 1. [file 41598_2023_27580_MOESM1_ESM.tif]

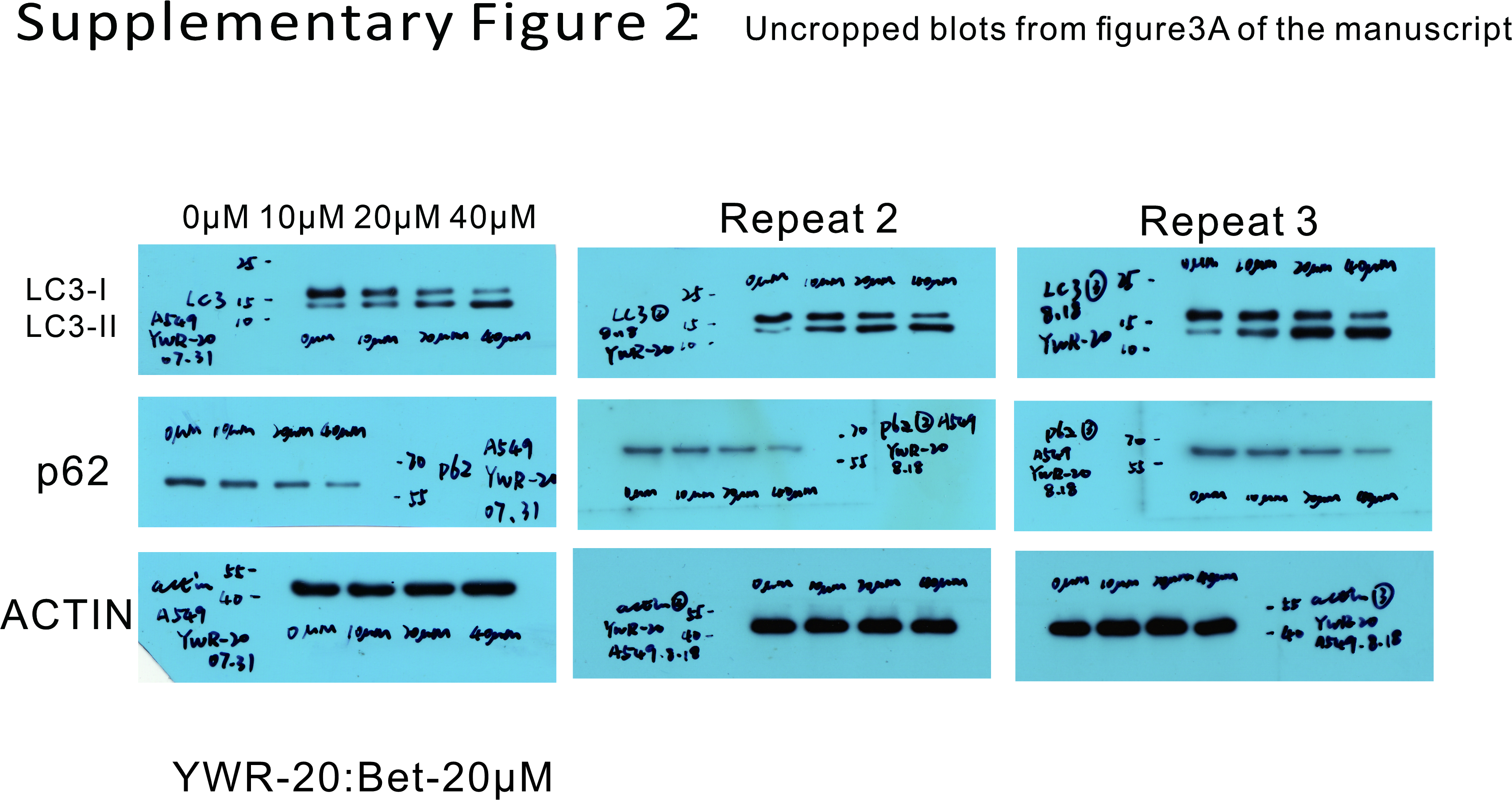

Supplement: Supplementary file 2 — Supplementary Figure 2. [file 41598_2023_27580_MOESM2_ESM.tif]

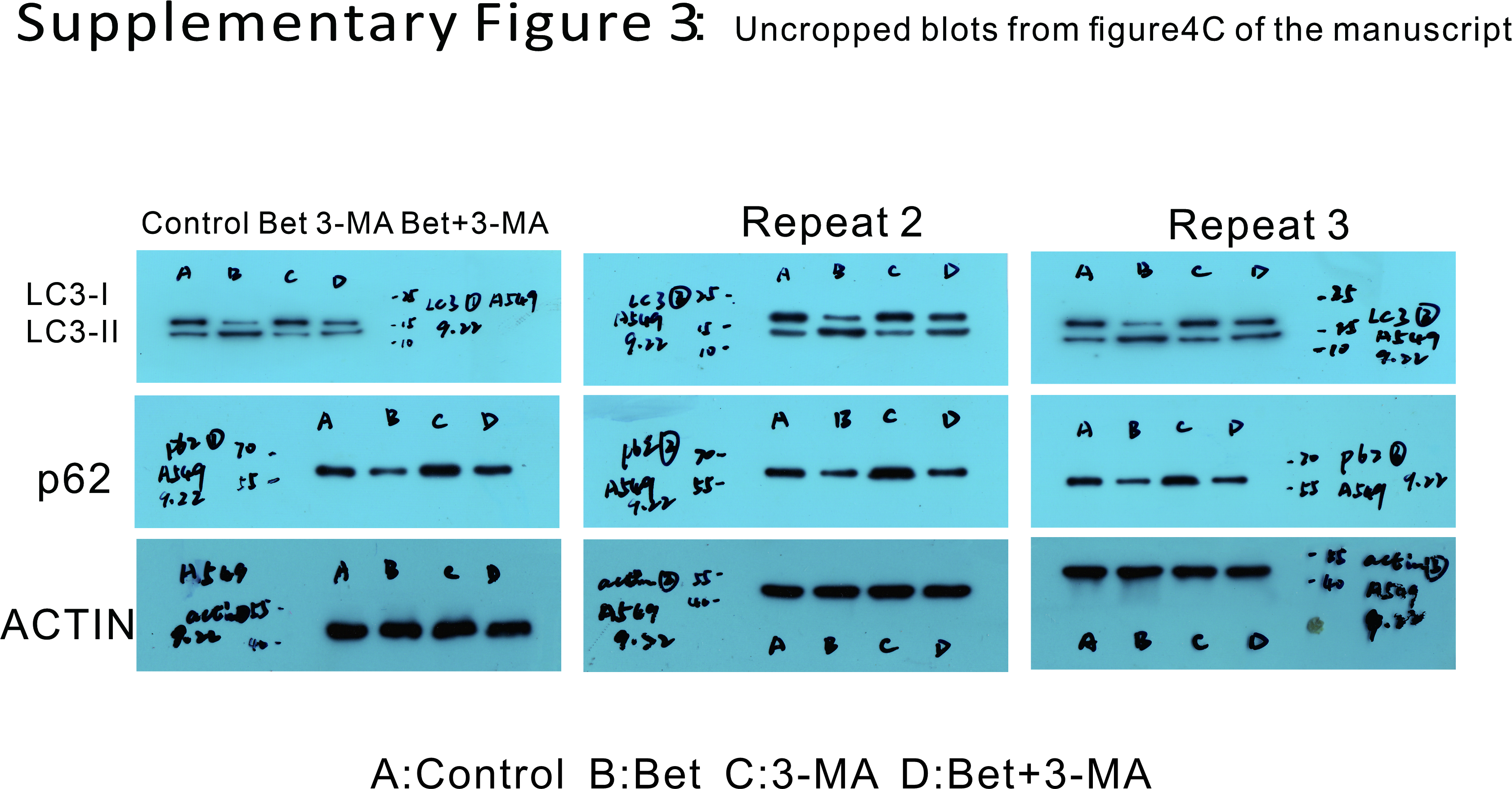

Supplement: Supplementary file 3 — Supplementary Figure 3. [file 41598_2023_27580_MOESM3_ESM.tif]

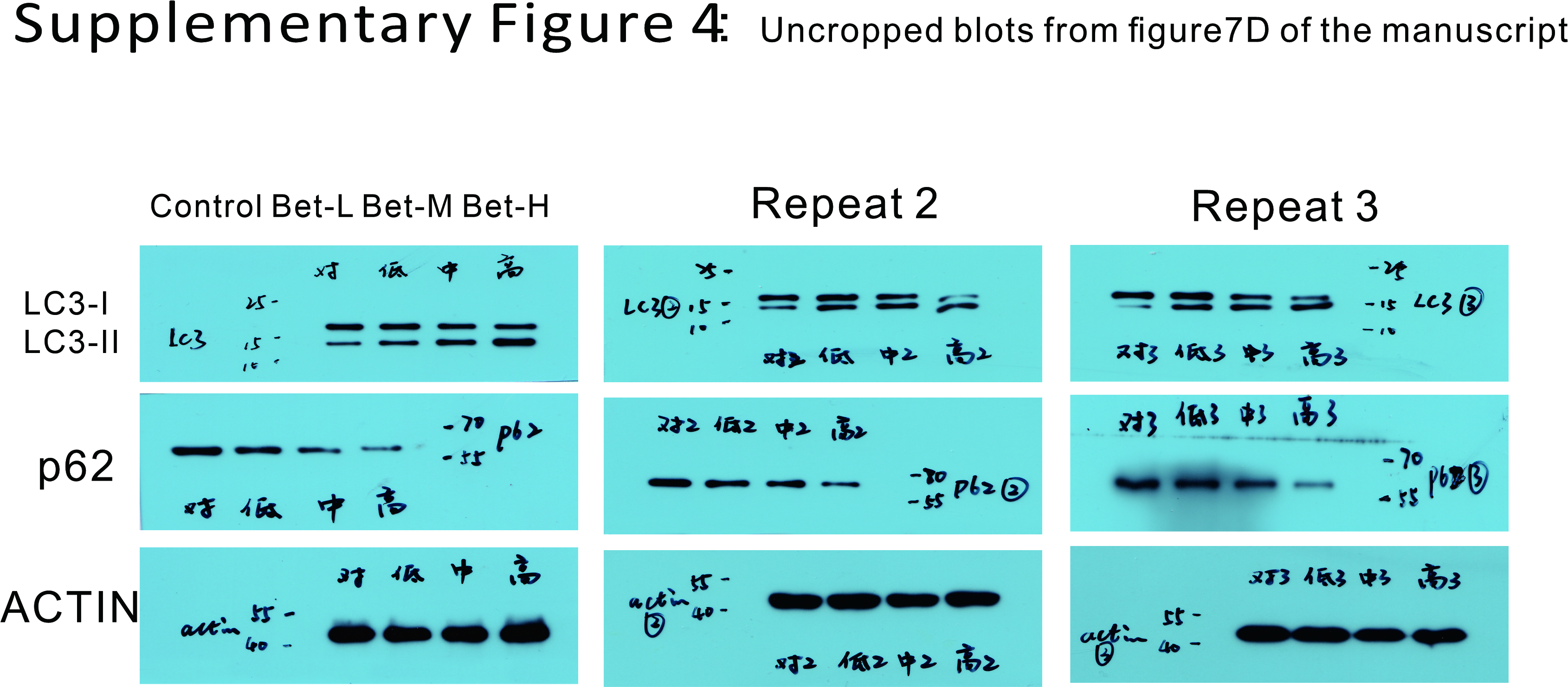

Supplement: Supplementary file 4 — Supplementary Figure 4. [file 41598_2023_27580_MOESM4_ESM.tif]
